# Exploring the Molecular Mechanism of Blue Flower Color Formation in *Hydrangea macrophylla* cv. “Forever Summer”

**DOI:** 10.3389/fpls.2021.585665

**Published:** 2021-02-17

**Authors:** Jiqing Peng, Xujie Dong, Chao Xue, Zhiming Liu, Fuxiang Cao

**Affiliations:** ^1^College of Life Science and Technology, Central South University of Forestry & Technology, Changsha, China; ^2^Department of Biology, Eastern New Mexico University, Portales, NM, United States; ^3^College of Landscape and Horticulture, Hunan Agricultural University, Changsha, China

**Keywords:** *Hydrangea macrophylla*, transcriptome, anthocyanins, carotenoids, flavonoids, flower color

## Abstract

*Hydrangea macrophylla* has a large inflorescence and rich colors, which has made it one of the most popular ornamental flowers worldwide. Thus far, the molecular mechanism of flower color formation in *H. macrophylla* flowers is unknown. By comparing the pigment content and transcriptome data of the bud period (FSF1), discoloration period (FSF2) and full-bloom stage (FSF3) of infertile blue flowers of *H. macrophylla* cv. “Forever Summer,” we found that genes associated with anthocyanin production were most associated with the formation of blue infertile flowers throughout development. The anthocyanin biosynthesis pathway is the main metabolic pathway associated with flower color formation, and the carotenoid biosynthesis pathway appeared to have almost no contribution to flower color. There was no competition between the flavonoid and flavonol and anthocyanin biosynthesis pathways for their substrate. At FSF1, the key genes *CHS* and *CHI* in the flavonoid biosynthesis pathway were up-regulated, underlying the accumulation of a substrate for anthocyanin synthesis. By FSF3, the downstream genes *F3H*, *C3′5′H*, *CYP75B1*, *DFR*, and *ANS* in the anthocyanin biosynthesis pathway were almost all up-regulated, likely promoting the synthesis and accumulation of anthocyanins and inducing the color change of infertile flowers. By analyzing protein–protein interaction networks and co-expression of transcription factors as well as differentially expressed structural genes related to anthocyanin synthesis, we identified negatively regulated transcription factors such as WER-like, MYB114, and WDR68. Their site of action may be the key gene *DFR* in the anthocyanin biosynthesis pathway. The potential regulatory mechanism of flower color formation may be that WER-like, MYB114, and WDR68 inhibit or promote the synthesis of anthocyanins by negatively regulating the expression of *DFR*. These results provide an important basis for studying the infertile flower color formation mechanism in *H. macrophylla* and the development of new cultivars with other colors.

## Introduction

*Hydrangea macrophylla* is an shrub in the family Saxifragaceae. As many different species and cultivars are widely used as cut flowers, as potted plants, and in landscaping because of their large inflorescences and beautiful colors, *H*. *macrophylla* has become one of the most promising ornamental flower species. Flower color has always been a focus of breeders and scientists, and it has been revealed that the formation of flower color is the result of the interactions between genes and the external environment (Kumar et al., 2008). Accordingly, the cultivation of blue *H. macrophylla* varieties can be achieved by altering external conditions; for example, changing soil pH or adding exogenous Al^3+^ can change the color of some infertile flowers of *H*. *macrophylla* (Yoshida et al., 2003; Eid, 2015; Hariri et al., 2015; Gong et al., 2017), but some varieties maintain a stable blue color under the same cultivation conditions. However, the molecular mechanism of the gradual blue color of the infertile flower formation process of *H*. *macrophylla* remains to be further studied.

Flower color is one of the most important ornamental traits in plants and plays an important role in improving plant quality. Plant color is mainly affected by anthocyanins (Zhao and Tao, 2015); their type and content are the most important factors affecting the formation of flower color (Dai, 2005; Han et al., 2008; Yamagishi et al., 2012). It has been found that the main substances affecting plant color are flavonoids and carotenoids among species in the order Caryophyllales (Nishihara and Nakatsuka, 2010; Brockington et al., 2015). Flavonoids are the main substances that determined the formation of most plant colors (Yoshioka et al., 2012). Among flavonoids, anthocyanins have the greatest influence on flower color. There are six main anthocyanins in plants: pelargonin, cyanidin, delphirin, paeoniflorin, paeoniflorin, and malvidin. Among them, peonidin is formed by methylation of cyanidin, and petunidin and malvidin are formed under different degrees of delphinium methylation (Martin et al., 1991; Hondo et al., 1992; Tanaka et al., 2009). Pelargonin appears brick red, while cyanidin and peonidin appear purple-red; delphinidin, petunidin, and malvidin are instead between purple and blue. Accordingly, these compounds can change the color of plants from pink to blue-violet (Kazuma et al., 2003; Wei et al., 2009). However, other flavonoids can cause the color of plants to exhibit varying degrees of yellowness. Carotenoids can make plants yellow, orange, and red (Kishimoto and Ohmiya, 2006; Chiou et al., 2010; Yamagishi et al., 2010a; Hai et al., 2012; Han et al., 2014). At present, the flavonoid biosynthesis pathway (Burns et al., 2013; Cheynier et al., 2013; Zhao and Tao, 2015) and carotenoid biosynthesis pathway are well understood (Yuan et al., 2015). The precursor of the flavonoid biosynthesis pathway is phenylalanine, which forms various types of anthocyanins after a three-step catalytic reaction. The first step is the conversion of phenylalanine to coumarin-CoA catalyzed by PAL, C4H, and 4CL; this step is a common pathway for the production of many secondary metabolites. The second step is the conversion of coumarate-CoA to dihydroflavonol under CHS, CHI, F3H, and F3′5′H. This is a key response in the metabolism of flavonoids. The third step is the formation of various stable anthocyanins under the catalysis of DFR, ANS, UFGT, and MT. The precursor of the carotenoid biosynthesis pathway is isoprenoid (Rodriguez-Concepcion, 2010), and many genes in this biosynthesis pathway have been studied (Farre et al., 2010; Kato, 2012; Ohmiya, 2013; Rodriguez-Concepcion and Stange, 2013; Liu L. et al., 2015). PSY/crtB, PDS, Z-ISO, ZDS, crtISO, crtZ, CCS1, ZEP, VDE, and NCED are key enzymes in the carotenoid biosynthesis pathway and play an important regulatory role in the accumulation of carotenoids. In addition to structural genes, transcription factors (including MYB, bHLH, and WD40) also have important regulatory effects on the accumulation of anthocyanins (Ramsay and Glover, 2005). The regulatory mechanisms by which transcription factors impact plant color have been verified in many plants (Hong et al., 2019; Fu et al., 2020), including petunia (Quattrocchio et al., 1993; Spelt et al., 2000; Albert et al., 2009, 2011), Japanese morning glory (Yasumasa et al., 2006), rose (Kui et al., 2010), Asiatic hybrid lily (Nakatsuka et al., 2009; Yamagishi et al., 2010b, 2012, 2014), chrysanthemum (Zhu et al., 2013; Liu X. et al., 2015), and phalaenopsis (Hsu et al., 2015), among others. MYB6 in Asiatic hybrid lily (Nakatsuka et al., 2009) and chrysanthemum (Liu X. et al., 2015) can change flower color by positively regulating a single structural gene, DFR. However, IpMYB1 in morning glory can alter flower color by regulating multiple structural genes (Yasumasa et al., 2006), and PhNYB27 in petunia can alter flower color by suppressing flavonoid genes (Albert et al., 2011). Thus, the regulatory mechanisms of transcription factors on plant color are diverse. The formation of plant flower color is affected by both structural genes and transcription factors.

Recently, transcriptome sequencing technology has been widely used in plant research owing to its low cost, speed, and efficiency (Wang et al., 2009, 2014; Loraine et al., 2013; Li et al., 2016). To reveal the molecular mechanisms influencing the development of blue flowers in *H. macrophylla*, transcriptome sequencing technology was used to analyze differentially expressed genes (DEGs) in infertile flowers across different developmental stages in the blue *H. macrophylla* cultivar “Forever Summer.” The biosynthesis pathways related to the accumulation of anthocyanidins, such as the flavonoid and carotenoid biosynthesis pathways, and flower-related transcription factors were also specifically examined. The purpose of this study was to provide guidance for the systematic investigation of the molecular mechanism of flower color formation.

## Materials and Methods

### Plant Materials

**FIGURE 1:**
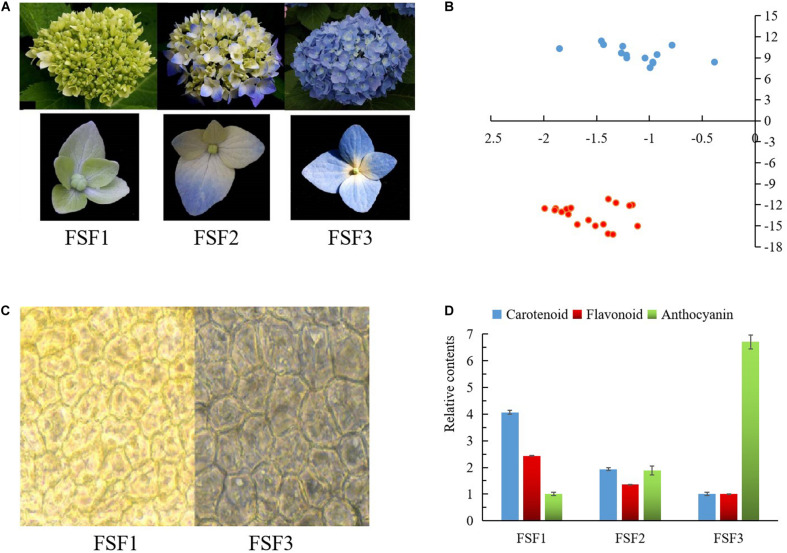
Images and difference analysis of infertile flower color at three developmental stages: FSF1, FSF2, FSF3. **(A)** Images of inflorescences and infertile flowers at these three stages. **(B)** Measured result of color testers at FSF1 and FSF3. Blue points represent the color values of infertile flowers at FSF1, and red points represent the color values of infertile flowers at FSF3. **(C)** Microscopic observation of epidermal cells from infertile flowers at FSF1 and FSF3. **(D)** The relative contents of anthocyanins, total flavonoids, and carotenoids in the infertile flower at the three developmental stages.

*Hydrangea macrophylla* cv. “Forever Summer” was planted in the Botanical Garden of the Central South University of Forestry and Technology, Changsha, Hunan, China, which has a soil pH of 6.8. At 10 am in April and May, three experimental samples for each developmental period were collected from three progenies obtained from cuttings of the same plant at the bud stage (FSF1), discoloration stage (FSF2), and full-bloom stage (FSF3). The samples were photographed during sampling, and the colors of the samples were compared and measured using colorimetric cards from the Royal Society of Landscape Architecture, a portable color difference meter, and a microscope (Figure 1A). In order to avoid RNA contamination during sampling, healthy infertile flowers with good growth and no visible contamination by pests or diseases were collected, rinsed three times with deionized water, immediately frozen in liquid nitrogen, and stored in a −80°C refrigerator.

### Determination of Relative Content of Pigment

#### Determination of Relative Content of Anthocyanins

For anthocyanin extraction, 0.2-g samples were ground into powder under liquid nitrogen, extracted in 10 mL of 1% acidic methanol solution under dark conditions at 4°C for 24 h, and then suspended by ultrasonication for 1 h. The supernatant was obtained after centrifugation at 10,000 rpm for 10 min and filtered through a 0.22-μm membrane filter. A UV-Vis spectrophotometer was used to read the absorbance at 530 nm. Three biological replicates were set up for each experiment. The trend in the relative content of anthocyanins across the three periods was calculated based on the absorbance value.

#### Determination of Relative Content of Flavonoids

For flavonoid extraction, 0.2-g samples were ground into powder under liquid nitrogen, extracted in 10 mL of methanol solution under dark conditions at 4°C for 24 h, and then suspended by ultrasonication for 1 h. The supernatant was obtained after centrifugation at 10,000 rpm for 10 min and filtered through a 0.22-μm membrane filter. Then, 2 mL of the supernatant was removed, and 2 mL of 1.5% AlCl_3_ solution and 3 mL of 1 M sodium acetate (pH 5.0) were added, thus keeping the volume at 10 mL. After 10 min, the absorbance was read at 415 nm using a UV-Vis spectrophotometer. Three biological replicates were set up for each experiment. The trend in the relative content of flavonoids across the three periods was calculated based on the absorbance values.

### Determination of Relative Content of Carotenoids

For carotenoid determination, 0.2-g samples were ground into powder under liquid nitrogen and extracted in 10 mL petroleum ether under dark conditions at 4°C for 24 h, and then suspended by ultrasonication for 1 h. The supernatant was obtained after centrifugation at 10,000 rpm for 10 min and filtered through a 0.22-μm membrane filter. The UV-Vis spectrophotometer was used to read the absorbance at 440 nm. Three biological replicates were set up for each experiment. The trend in the relative content of carotenoids across three periods was calculated based on the absorbance values.

### Transcriptome Sequencing and Data Analysis

Transcriptome sequencing of infertile flowers from *H*. *macrophylla* cv. “Forever Summer” at three different flowering periods was performed by Beijing Nuohe Zhiyuan Biotechnology Co., Ltd. Clean reads were assembled (Grabherr et al., 2011) and annotated, and plant transcription factors were predicted using iTAK software (Zheng et al., 2016). To analyze the expression level of genes (Trapnell et al., 2010), RSEM software (Li and Dewey, 2011) was used to analyze the number of read counts for each gene. The parameters used in bowtie 2 took their default values, and fragments per kilobase of exon model per million mapped reads (FPKM) conversion was then performed. The DESeq R package was used for gene differential expression analysis, and the screening threshold was *p*_adj_ < 0.05 (Anders and Huber, 2010). A Venn diagram of DEGs was drawn based on these results, and the DEGs were also analyzed by Kyoto Encyclopedia of Genes and Genomes (KEGG) classification and KEGG enrichment Using KOBAS2.0 (Xie et al., 2011).

### Gene Validation and Expression Analysis

To verify the accuracy of the transcriptome data, four unigenes related to anthocyanin synthesis were selected for qPCR analysis, and *Actin* was selected as an internal reference gene. Specific primers were designed using primer software version 5 (Supplementary Table 1). The qPCR reaction system was prepared according to the manufacturer’s instructions for the 2 × SYBR Green Master Mix Enzyme kit (Biotool, Houston, TX, United States). PCR amplification proceeded as follows: predenaturation at 95°C for 5 min, 40 cycles of 95°C for 15 s and annealing at 60°C for 40 s. Dissolution curves were recorded from 60°C to 95°C, with a 0.5°C increase every 5 s. Each reaction was repeated three times. The relative expression level of the target genes was calculated by the 2^−ΔΔCq^ method. Correlation analysis was performed using SPSS version 17.0 software (SPSS Inc., Chicago, IL, United States) according to the relative expression of the gene and its FKPM value.

### Screening of Key Structural Genes in Pigment Synthesis Pathways

Based on the FPKM values of the DEGs in the flavonoid and carotenoid biosynthesis pathways, a heat map of the three flowering periods was drawn using the pheatmap package in the R statistical computing environment. Then, an expression map of the DEGs was drawn according to the KEGG pathway map. Based on the expression map, the expression rules were comprehensively analyzed, and the key genes related to pigment substance synthesis were screened.

### Screening of Key Transcription Factors During Flower Formation

The transcription factor expression data, which included expression levels for MYB, bHLH, WD40, and the DEGs identified in the flavonoid biosynthetic pathway, was screened using blastx software, with an e-value of 1e-10. The target gene set sequence was aligned to the protein sequence of the reference species contained in the string database^1^, and the protein interaction relationship of the reference species was used to construct an interaction network. Network visualization for the interaction network related to MYB and DEGs was performed using Cytoscape version 3.6.1. A phylogenetic tree of MYB transcription factors and WD from different species was constructed using the maximum likelihood method with 1000 bootstrap replicates with MEGA version 6.0 (Koichiro et al., 2013). Co-expression analysis was performed using SPSS 17.0 based on the transcription factor and DEG data.

## Results

### Pigment Levels in Infertile Flowers at Three Developmental Stages

During the development of the blue infertile flowers of *H*. *macrophylla* cv. “Forever Summer,” the color changed from yellow-green to bright blue-violet (Figure 1A). According to the colorimetric card from the Royal Society of Landscape Architecture, the infertile flowers at FSF1 were yellow-green (RHS 150C), the distal sepals and proximal sepals of infertile flower at FSF2 were purple-blue (RHS 100D) and yellow-green (RHS 150C), respectively, and the infertile flowers at FSF3 were bright blue-violet (RHS 98C). The color values of FSF1 and FSF3 infertile flowers were distributed in the second and the third quadrant respectively according to color testers (Figure 1B). The color of the epithelial cells at FSF3 was significantly different from that at FSF1, and blue matter was accumulated in the epithelial cells by FSF3 (Figure 1C). As shown in Figure 1D, the carotenoid contents and the flavonoid contents decreased from FSF1 to FSF3, and the anthocyanin contents increased. This indicates that the blue infertile flower color formation process of *H. macrophylla* cv. ‘Forever Summer’ is associated with the reduction of flavonoids and carotenoids, as well as an increase in anthocyanins.

### Library Construction and Transcriptome Sequencing

**FIGURE 2:**
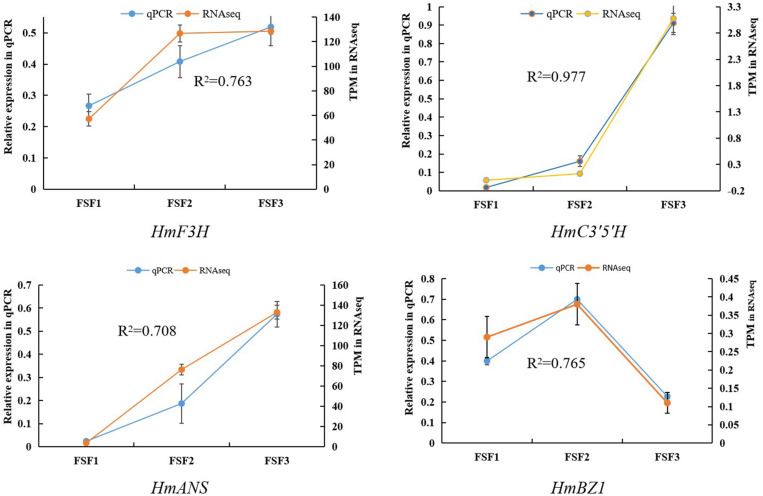
Correlation analysis between qRT-PCR and RNA-seq results for *HmF3H*, *HmC3′5′H*, *HmANS*, and *HmBZ1*.

To understand the molecular basis of the blue infertile flower color change in *H. macrophylla* cv. “Forever Summer,” infertile flowers at the FSF1, FSF2, and FSF3 stages were used to construct three libraries for high-throughput sequencing. Thus, 69,281,730, 62,700,144, and 73,712,440 raw reads were obtained from the FSF1, FSF2, and FSF3 sequencing libraries, respectively. After removal of adaptor sequences, ambiguous reads, and low-quality reads, 66,658,800, 60,767,008, and 70,675,124 high-quality clean reads comprising 9.99, 9.12, and 10.60 Gb with Q20 >96.90% were obtained from FSF1, FSF2, and FSF3 transcriptome sequencing, respectively. The correlation coefficient of gene expression levels between the three biological replicate samples of the infertile flowers exceeded 0.81. To verify the accuracy of the transcriptome data, a correlation analysis was performed based on transcripts per million (TPM) as a measure of transcript abundance, and the relative expression of four representative genes was also analyzed by qPCR. The relative expression levels of qPCR for the four genes (*HmF3H*, *HmC3′5′H*, *HmANS*, *HmBZ1*) were closely related to their FPKM values. The Pearson correlation coefficients between the two estimates of the *HmF3H*, *HmC3′5′H*, *HmANS*, and *HmBZ1* expression levels were 0.76, 0.98, 0.71, and 0.77, respectively, and significant at a *P* < 0.05 threshold (Figure 2). All raw high throughput sequence data have been deposited in the NCBI SRA database under accession number PRJNA588557.

### Identification and Analysis of DEGs Among Infertile Flowers at Three Stages

**FIGURE 3:**
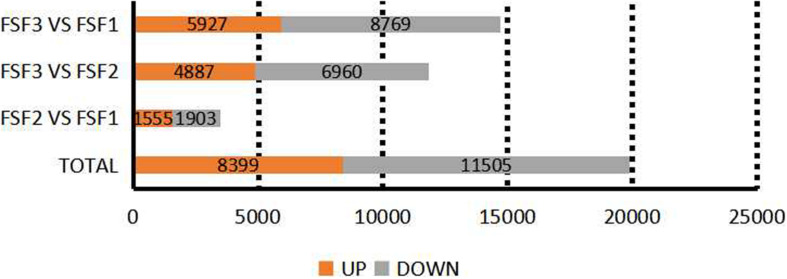
Statistics on the number of differentially expressed genes among three development stages of infertile flowers of *Hydrangea macrophylla* cv. “Forever Summer.”

According to the read count data obtained from the gene expression level analysis, DESeq (Anders and Huber, 2010) was used to screen the transcripts for differential expression based on a negative binomial distribution, at a *p*_adj_ < 0.05 level. A total of 19,250 DEGs were thus obtained (Figure 3). In total, 3458 DEGs were found between FSF1 and FSF2, with 1555 unigenes up-regulated and 1,903 unigenes down-regulated. There were 11,847 DEGs between FSF2 and FSF3, with 4,887 DEGs up-regulated and 6,960 DEGs down-regulated. The number of DEGs between FSF3 and FSF1 was the highest, with a total of 14,696, of which 5,927 were up-regulated and 8,769 were down-regulated. The number of DEGs increased fastest over time from FSF2 to FSF3, and the highest number of DEGs was between FSF1 and FSF3.

**FIGURE 4:**
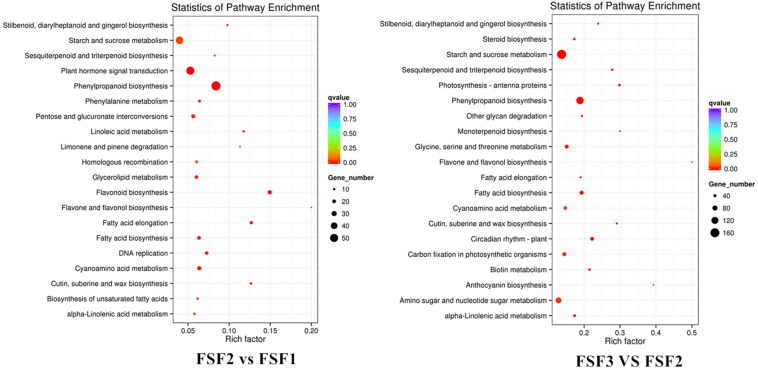
Kyoto Encyclopedia of Genes and Genomes (KEGG) enrichment analysis of infertile flower of *Hydrangea macrophylla* cv. “Forever Summer” at the FSF1, FSF2, and FSF3 developmental stages.

Kyoto Encyclopedia of Genes and Genomes pathway enrichment analysis was performed on the identified DEGs. The numbers of DEGs enriched among KEGG pathways were 987, 3531, and 4035, respectively, which were attributed to 105, 121, and 120 metabolic pathways in FSF1, FSF2, and FSF3, respectively. The top 20 enriched metabolic pathways were explored (Figure 4). Flavone and flavonol biosynthesis, phenylpropanoid biosynthesis, and flavonoid biosynthesis were enriched in the FSF2 versus FSF1 comparison. Flavone and flavonol biosynthesis, phenylpropanoid biosynthesis, and anthocyanin biosynthesis were enriched in the FSF3 versus FSF2 comparison. During the development of the blue infertile flowers, flavone and flavonol biosynthesis and phenylpropanoid biosynthesis were always significantly different. Flavonoid biosynthesis was significantly enriched in the FSF2 versus FSF1 comparison, while anthocyanin biosynthesis was significantly enriched in the FSF3 versus FSF2 comparison; thus, anthocyanin biosynthesis might be more important than flavonoid biosynthesis for formation of blue coloration of infertile flowers. In addition, the other two metabolic pathways involved in flower color formation, including the carotenoid and isoflavone biosynthesis pathways, were also found in the KEGG enrichment pathway. The difference in these metabolic pathways may underlie the formation of the blue coloration of infertile flowers.

### Analysis of DEGs in the Flavonoid Biosynthesis Pathway

**FIGURE 5:**
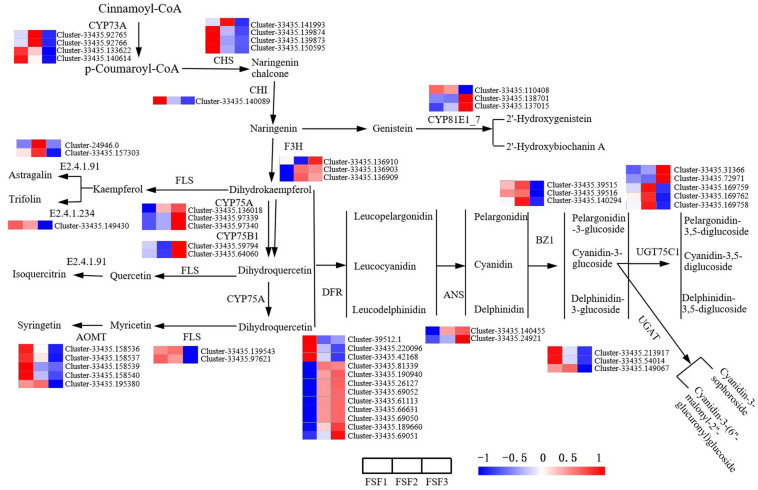
Analysis of differentially expressed gene in the flavonoid biosynthesis pathway for infertile flowers of *Hydrangea macrophylla* cv. “Forever Summer” at the FSF1, FSF2, and FSF3 developmental stages. The color scale represents log2-transformed FPKM (fragments per kilobase of exon per million mapped reads) values. Red represents high expression, and blue represents low expression.

Flavone, flavonol, anthocyanin, and isoflavone biosynthesis pathways are branched pathways of flavonoid biosynthesis, which can be incorporated into flavonoid biosynthesis for analysis. The direct prerequisites for the flavonoid biosynthesis pathway are cinnamoyl-CoA and *p*-coumaroyl CoA. Proanthocyanidins are formed in the catalysis of CYP73A, CHS, CHI, F3H, CYP75A, CYP75B1, and DFR. In the main pathway of the flavonoid biosynthesis pathway, a total of 29 DEGs were obtained, involving seven enzymes. Except for the two enzymes CHI and LAR, the other structural genes exist in multiple copies. As shown in Figure 5, the expression levels of DEGs encoding the enzymes CYP73A, CHS, and CHI were up-regulated during FSF1 and FSF2, but down-regulated during FSF3. As the rate-limiting enzyme in the flavonoid biosynthesis pathway, CHS and CHI have important effects on the accumulation of flavonoids. These genes are highly expressed during FSF1 and FSF2, which may be related to the accumulation of flavonoids during these periods. At FSF3, the expression levels of the DEGs encoding F3H, DFR, CYP75A, and CYP75B1 were almost all up-regulated, which may be related to the rapid accumulation of anthocyanidins in this period.

Proanthocyanidins are precursors in the anthocyanin biosynthesis pathway and are converted into various stable anthocyanins under the catalysis of enzymes that include ANS, BZ1, UGAT, and UGT75C1. In the anthocyanin biosynthesis pathway, 13 DEGs encoding the enzymes BZ1, UGAT, and UGT75C1 were identified. ANS is a key enzyme in the anthocyanin biosynthesis pathway and plays an important regulatory role in anthocyanin accumulation. The two DEGs encoding ANS were significantly up-regulated at FSF3, which is consistent with the color change during this period. In the flavonoid and flavonol biosynthesis pathway, 10 DEGs encoding FLS, E2.4.1.91, E2.4.1.234, and AOMT were obtained. All DEGs were down-regulated during FSF3.

### Analysis of DEGs in the Carotenoid Biosynthesis Pathway

**FIGURE 6:**
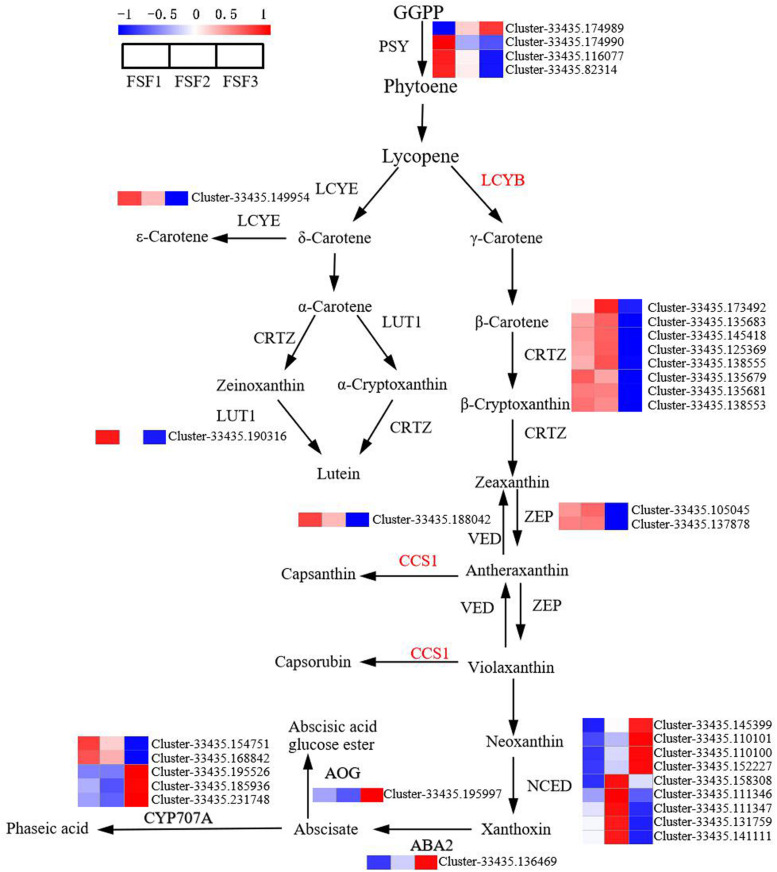
Analysis of differentially expressed genes in the carotenoid biosynthesis pathway in infertile flowers of *Hydrangea macrophylla* cv. “Forever Summer” at the FSF1, FSF2, and FSF3 developmental stages. The color scale represents log2-transformed FPKM (fragments per kilobase of exon per million mapped reads) values. Red represents high expression, and blue represents low expression.

Carotenoids also have an important influence on plant color formation. In order to study the role of structural genes in the carotenoid biosynthesis pathway on flower color formation, DEGs were analyzed at the FSF1, FSF2, and FSF3 stages. A total of 33 DEGs were obtained in these three periods (Figure 6). From geranylgeranyl pyrophosphate (GGPP) to lutein and neoxanthin in the pathway diagram in Figure 6, 17 DEGs encoding six enzymes were found, and almost all DEGs were down-regulated from FSF1 to FSF3. In *H*. *macrophylla* cv. “Forever Summer,” the decomposition pathway of carotenoids was the BCH pathway (Meng et al., 2013), and the final product was abscisic acid. In the process of carotenoid decomposition, 14 of the 16 DEGs encoding four enzymes were up-regulated at FSF2 or FSF3. The genes involved in carotenoid synthesis were down-regulated, but genes involved in carotenoid breakdown were up-regulated, likely leading to a decrease in carotenoids during the flowering process.

### Identification of Transcription Factors

**FIGURE 7:**
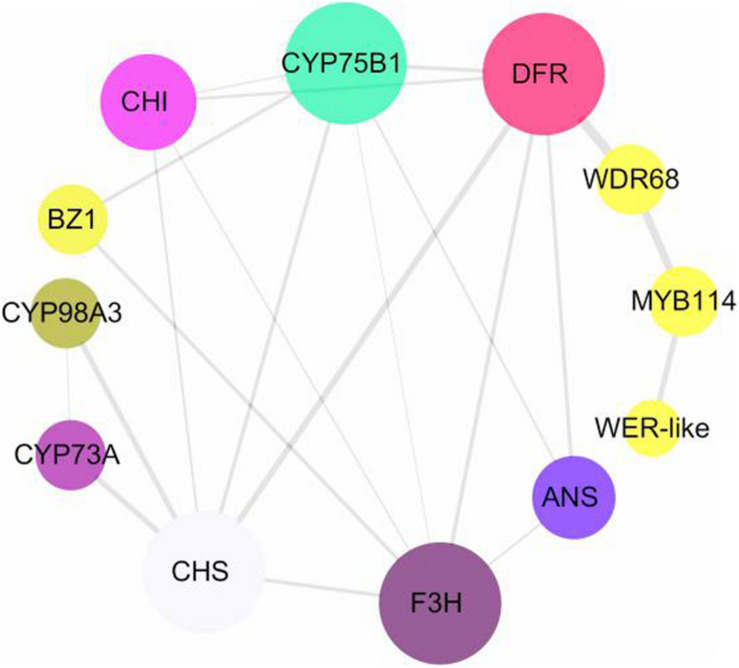
Protein–protein interaction network of putative genes related to flavonoid biosynthesis.

To screen for key transcription factors involved in anthocyanin synthesis, transcription factors and structural genes were analyzed using interaction networks. We found that two MYB transcription factors and one WDR68 (including three unigenes) participated in the regulation of structural genes in the anthocyanin biosynthesis pathway (Figure 7). The phylogenetic relationships among MYB and WD transcription factors from different species showed that MYB114 and WER-like proteins in *Hydrangea* are closely related to the homologous transcription factors in *Vitis vinifera*, *Prunus avium*, and *Malus domestica*, and the WD transcription factor in *Hydrangea* is also closely related to the homologous transcription factors in *Camellia sinensis* and tree peony (Supplementary Figure 1). CsWD40 and PsWD40 regulate anthocyanin biosynthesis and accumulation in the *C*. *sinensis* and tree peony, respectively (Zhang et al., 2014; Wang et al., 2019). Therefore, WDR68 in *Hydrangea* may also regulate anthocyanin synthesis. According to the FPKM values of the transcription factors and structural genes, their Pearson correlation coefficients were calculated using SPSS version 17.0. The FPKM values of the unigenes of the MYB transcription factor and the two unigenes of the WDR68 (Cluster-33435.149152 and Cluster-33435.158759) were positively correlated (*P* < 0.01) and negatively correlated with the *F3H*, *DFR*, and *ANS* FPKM values (*P* < 0.01), respectively. Additionally, the FPKM value of a WDR68 unigene (Cluster-33435.149152) was negatively correlated with those of *F3H* and *DFR* (*P* < 0.01), respectively (Table 1). MYB transcription factors, including WER-like, MYB114, and WDR68 (Cluster-33435.149152 and Cluster-33435.158759) might have an important influence on the formation of the blue coloration of infertile flowers in *Hydrangea*.

**TABLE 1:** Co-expression of putative genes related to flavonoid biosynthesis.

Gene name	#ID	Cluster-33435.149151	Cluster-33435.149152	Cluster-33435.158759	*CHS*	*F3H*	*C3′5′H*	*CYP75B1*	*DFR*	*ANS*	*BZ1*
*WER-like*	Cluster-33435.54542	0.49	0.861**	0.904**	–0.082	−0.857**	–0.598	–0.395	−0.830**	−0.849**	0.222
*MYB114*	Cluster-33435.66205	0.606	0.805**	0.823**	0.064	−0.839**	−0.698*	–0.546	−0.805**	−0.893**	0.318
*WDR68*	Cluster-33435.149151	1	0.253	0.463	0.392	–0.500	−0.683*	–0.564	–0.483	−0.670*	0.608
*WDR68*	Cluster-33435.149152	0.253	1	0.779*	–0.252	−0.853**	–0.537	–0.301	−0.862**	−0.767*	0.052
*WDR68*	Cluster-33435.158759	0.463	0.779*	1	–0.195	−0.706*	–0.349	–0.110	−0.724*	–0.647	0.219

** represent significant at a *P* < 0.01 threshold, * represents significant at a *P* < 0.05 threshold.

## Discussion

### Flavonoid Biosynthesis Pathway Changes Are Associated With Blue Infertile Flower Development

The formation of plant flower color is mainly affected by the accumulation of flavonoids and carotenoids. Anthocyanins are water-soluble flavonoids responsible for the red, pink, blue, and purple coloration of flowers (Han et al., 2008). In infertile flowers of the *H*. *macrophylla* cv. “Forever Summer,” blue anthocyanin is accumulated in the upper epidermal cells, and the total anthocyanin content was significantly increased by FSF3. However, the total flavonoid and carotenoid contents decreased during the development of infertile flowers. Furthermore, based on KEGG pathway enrichment analysis, anthocyanin biosynthesis was enriched in the FSF3 versus FSF2 and FSF3 versus FSF1 comparisons. This suggested that the accumulation of anthocyanins is the main factor causing infertile flowers to turn blue.

The anthocyanin biosynthesis pathway is a branch of the flavonoid biosynthetic pathway. The synthesis and accumulation of anthocyanins are affected by flavonoids. In the flavonoid biosynthetic pathway, *CYP73A*, *CHS*, and *CHI* are upstream genes, while *F3H*, *C3′5′H*, *CYP75B1*, *DFR*, and *ANS* are downstream. They encode key enzymes in the flavonoid biosynthetic pathway (Zhao and Tao, 2015) and thus play an important role in the development of flower color. CHS and CHI have important effects on the accumulation of flavonoids. A previous study reported that in untransformed tobacco flowers, anthocyanin was not accumulated, while the accumulation of flavonoids increased by over-expression of *CHI* from chrysanthemum (Li et al., 2006). Overexpression of peony *CHI* in tobacco also increased the accumulation of flavonoids (Zhou et al., 2014). During the development of infertile flowers of *H*. *macrophylla* cv. “Forever Summer,” unigenes encoding CHS and CHI were almost all up-regulated and expressed at FSF1, and the flavonoid content was highest in this period. Up-regulated expression of early expressed genes in the flavonoid biosynthetic pathway may cause accumulation of flavonoid and provide precursors for anthocyanin synthesis. F3H catalyzes naringenin into dihydroflavones. Three types of dihydroflavones (dihydromyricetin, dihydroquercetin, and dihydrokaempferol) are reduced in the presence of DFR and NADPH. The level of DFR expression can cause flower color change, and *DFR* has the highest expression in petals that accumulate large amounts of anthocyanins (Nakatsuka et al., 2003; Zhao et al., 2012). C3′5′H is a key enzyme involved in the synthesis of delphinidin, which can promote the formation of blue flowers (Tanaka and Brugliera, 2013). ANS can catalyze the conversion of proanthocyanidins into colored anthocyanins, which is the last key enzyme in the flavonoid biosynthetic pathway. Deletion of the ANS gene sequence can reduce the content of anthocyanins (Shimizu et al., 2011). During the formation of the *H*. *macrophylla* cv. “Forever Summer” infertile blue flowers, unigenes of the downstream genes *F3H, C3′5′H, CYP75B1, DFR*, and *ANS* in the flavonoid biosynthetic pathway were almost all up-regulated. This expression trend is consistent with the trend in anthocyanin content, so the flavonoid biosynthetic pathway is likely the key metabolic pathway involved in the formation of the blue coloration of infertile flowers of *H*. *macrophylla* cv. “Forever Summer.” *F3H, C3′5′H, CYP75B1, DFR*, and *ANS* may be the main factors underlying the formation of the blue coloration of the infertile flowers. Additionally, the flavonoid and flavonol biosynthetic pathways are branched pathways of the flavonoid biosynthetic pathway and share the same substrate as the anthocyanin biosynthetic pathway. Therefore, they should compete for the same substrate. However, during FSF3, the expression levels of DEGs in the flavonoid and flavonol biosynthetic pathways were all down-regulated and did not show a competitive effect on the substrate for anthocyanin synthesis.

### Carotenoid Biosynthesis Pathway Is Not a Key Metabolic Pathway Involved in the Formation of Blue Infertile Flowers

Carotenoids are also the basis for the formation of flower coloration in many plants, mainly underlying yellow, orange, and red coloration. The flower color of plants, such as Chinese narcissus (Ren et al., 2017) and *Camellia* (Zhou et al., 2017), is mainly affected by carotenoids, and its regulatory mechanism has also been well elucidated. During the formation of infertile flowers, almost all DEGs in the carotenoid biosynthetic pathway, including *PSY, CRTZ, ZEP*, and *VED*, were up-regulated at FSF1 and FSF2, and the high expression of these genes likely increased carotenoid content. However, at FSF3, the DEGs *PSY, CRTZ, ZEP*, and *VED* were all down-regulated, while *ABA2, AOG*, and *CYP707A* were up-regulated, which likely accelerated the metabolism of carotenoids. This explains why the total amount of carotenoids gradually decreases throughout the development of infertile flowers. It also indicates that the carotenoid biosynthesis pathway has a relatively small contribution on the formation of blue infertile flowers of the hydrangea variety “Forever Summer.”

### MER-like, MYB114, and WDR68 May Be Key Negative Regulatory Transcription Factors of Flower Color Formation

The transcription factors MYB, bHLH, and WDR have important regulatory effects on the formation of flower color in plants (Ramsay and Glover, 2005). Thus far, many transcription factors have been discovered. PhAN2 (Quattrocchio et al., 1993), PhAN4 (Albert et al., 2009), PhPHZ (Albert et al., 2011), PhDPL (Spelt et al., 2000), and PhMYB27 (Albert et al., 2011) were found in petunia. LrMYB15, a transcription factor that regulates *CHSa, CHSb, DFR*, and *ANS*, was found in lily (Yamagishi, 2016). CmMYB6 and CmMYB1 were found in chrysanthemums (Zhu et al., 2013; Liu X. et al., 2015; Hong et al., 2019). The transcription factors PeMYB2, PeMYB11, and PeMYB12 have been found in *Phalaenopsis* (Hsu et al., 2015). Transcription factors can individually or cooperatively regulate structural genes, and the regulation can be positive or negative. In short, transcription factors regulate flower color in a variety of ways. In blue infertile flowers of *H*. *macrophylla* cv. “Forever Summer,” DEGs and transcription factors were analyzed based on their network interactions, phylogenetic relationships, and co-expression. The regulation of MYB transcription factors did not directly affect structural genes, but may instead regulated the synthesis of anthocyanins through WDR68. The key structural gene in the flavonoid biosynthesis pathway is *DFR*. The transcription factors CmMYB6, LhMYB6, and RhMYB10, which act on *DFR*, have been found in chrysanthemum (Liu X. et al., 2015), Asian lily (Nakatsuka et al., 2009), rose (Kui et al., 2010), and other plants. MER-like, MYB114, and WDR68 negatively regulate *DFR*. A possible regulatory mechanism involves the transcription factors WER-like, MYB114, and WDR68 being highly expressed at FSF1, thus inhibiting the synthesis of anthocyanins by regulating the expression of *DFR.* During FSF3 and FSF2, however, the expression levels of MYB and WDR decreased, and the inhibitory effect on DFR was thus weakened. This in turn led to the rapid synthesis and accumulation of a high level of anthocyanins, which accelerated infertile *H*. *macrophylla* “Forever Summer” turning blue.

## Conclusion

This study shows that the formation of blue infertile flowers of *H*. *macrophylla* cv. “Forever Summer” is mainly affected by anthocyanin accumulation. *DFR* is a key gene in the anthocyanin biosynthesis pathway. WER-like, MYB114, and WDR68 may be the key transcription factors regulate the synthesis of anthocyanins by negatively regulating *DFR*, which appears to affect the color of infertile flowers of *H*. *macrophylla* cv. “Forever Summer.”

## Data Availability Statement

The datasets presented in this study can be found in online repositories. The names of the repository/repositories and accession number(s) can be found below: https://www.ncbi.nlm.nih.gov/, PRJNA588557.

## Author Contributions

JP was responsible for the data analysis and drafted the manuscript. XD and CX assisted with the data analysis. ZL provided helpful comments on the manuscript. FC provided guidance on the whole study and contributed with valuable discussions. All authors read and approved the final manuscript.

## Conflict of Interest

The authors declare that the research was conducted in the absence of any commercial or financial relationships that could be construed as a potential conflict of interest.

## Footnotes

1 http://string-db.org/
