# Supplementary figures and images for: Exploring the Molecular Mechanism of Blue Flower Color Formation in Hydrangea macrophylla cv. “Forever Summer”
Source: Front Plant Sci. 2021 Feb 17;12:585665. doi: 10.3389/fpls.2021.585665 (PMC7925886; doi:10.3389/fpls.2021.585665)

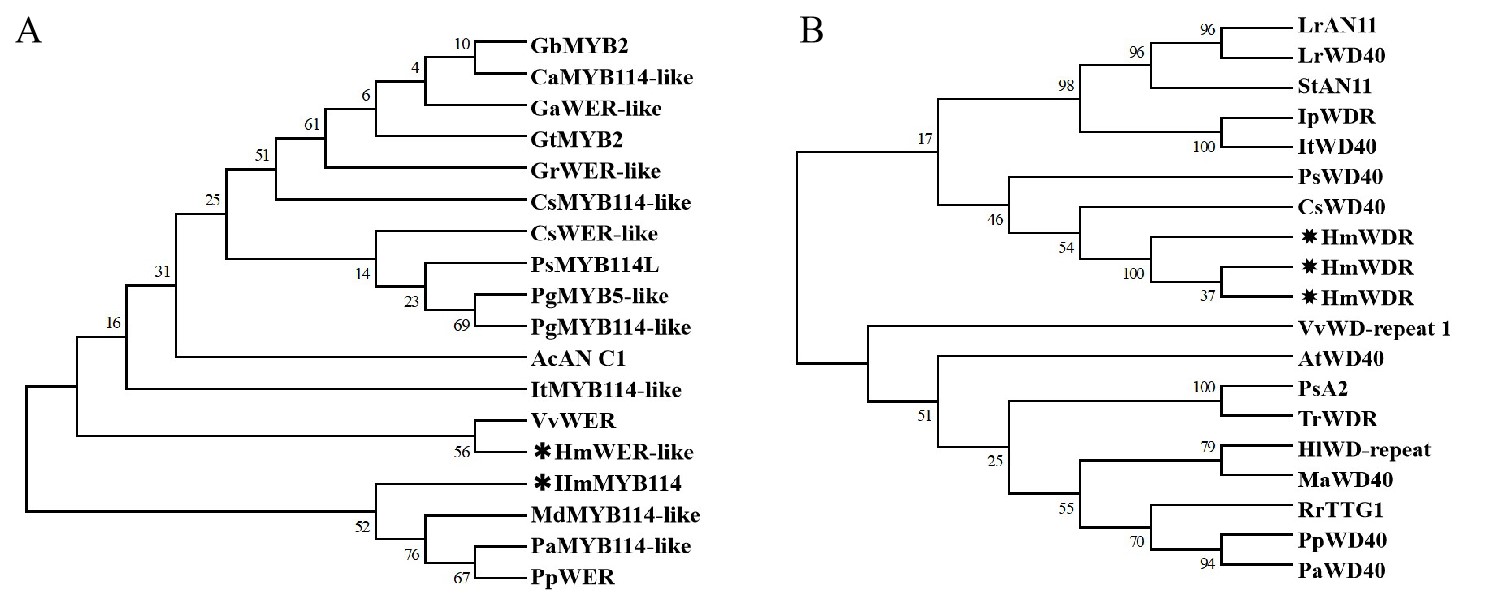

Supplement: Supplementary file 1 [file Image_1.JPEG]
