# Supplementary material for: Exploring the Molecular Mechanism of Blue Flower Color Formation in Hydrangea macrophylla cv. “Forever Summer”
Source: Front Plant Sci. 2021 Feb 17;12:585665. doi: 10.3389/fpls.2021.585665 (PMC7925886; doi:10.3389/fpls.2021.585665)
Supplement: Supplementary file 2 [file Table_1.doc]

Supplementary Table 1. Sequences of primers used in this study

| Primer | Sequence |
| --- | --- |
| QHmF3H-F | 5′ACAGCTCTGGCGGAGGAGAAG3′ |
| QHmF3H-R | 5′CGTCGCTGAACTGGTTGTAGGC3′ |
| QHmC3'5'H-F | 5′AGAGGATTGGGCTCCCATTCGG3′ |
| QHmC3'5'H-R | 5′GGCACAACCACTAGCTCACACC3′ |
| QHmANS-F | 5′GCCACGAGCGAGTATGCGAAG3′ |
| QHmANS-R | 5′CCAGCCTTCCTTCTTCCAAGCC3′ |
| QHmBZ1-F | 5′CGGTGGAGGCGGAGATCGG3′ |
| QHmBZ1-R | 5′CAGGGAGCAAGCACCAGAAGTC3′ |
